# Implementation of monocular near-infrared camera tracking in MRI and CT navigation systems

**DOI:** 10.1007/s11548-026-03758-1

**Published:** 2026-07-30

**Authors:** Javier Moviglia, Lukas Kuntz, Florian Kehrein, Urs Florian Herrmann, Jan Stallkamp, Marius Siegfarth

**Affiliations:** Mannheim Institute for Intelligent Systems in Medicine (MIISM), Heidelberg University, Mannheim, Germany

**Keywords:** Navigation system, Image-guided intervention, Percutaneous biopsy, Optical tracking, MRI, CT

## Abstract

****Purpose**:**

Image-guided intervention (IGI) systems support anatomical registration and real-time instrument tracking during minimally invasive procedures. Existing robotic and navigation solutions are often single-modality, costly, or have limited MRI compatibility. This study presents a cost-effective monocular near-infrared (NIR) tracking system for IGI across multiple modalities while maintaining accuracy and workflow efficiency.

****Methods**:**

The system, using retroreflective ArUco fiducial markers, was applied to phantom-based percutaneous liver biopsies. Two experiments were conducted under computed tomography (CT) and MRI guidance. Real-time tracking of the needle relative to the phantom and imaging data was performed. An interactive navigation interface was implemented in 3D Slicer. Targeting accuracy and procedure times were evaluated quantitatively.

****Results**:**

In the CT-guided study, the target positioning error (TPE) and needle-tip tracking error (TTE) were $10.3 \pm 3.7\,\textrm{mm}$ and $5.0 \pm 1.3\,\textrm{mm}$, respectively, with a total procedure time of $62 \pm 34\,\textrm{s}$. In the MRI-guided study, the corresponding values were $4.6 \pm 1.0\,\textrm{mm}$, $4.7 \pm 2.3\,\textrm{mm}$, and $113 \pm 65\,\textrm{s}$, respectively.

****Conclusion**:**

The proposed navigation system demonstrates strong potential as an accessible and versatile alternative for image-guided needle interventions across multiple imaging modalities. Furthermore, the system shows promise for future extension to other IGI procedures.

**Supplementary Information:**

The online version contains supplementary material available at https://doi.org/10.1007/s11548-026-03758-1.

## Introduction

Image-Guided Intervention (IGI) systems support minimally invasive procedures by integrating preoperative and intraoperative imaging for planning, guidance, and execution. By establishing a geometric relationship between patient anatomy, imaging data, and interventional instruments, IGI enables instrument localization across a wide range of applications, including vascular, thoracic, abdominal, neurointerventional, and percutaneous procedures [1]. Common modalities include CT, MRI, fluoroscopy, and ultrasound, used alone or in combination depending on the clinical task.

IGI systems can be broadly classified according to how image-to-patient correspondence is achieved [1]. In direct visualization approaches, both anatomy and instruments are imaged simultaneously, while navigation-based systems rely on geometric registration and real-time tracking to localize instruments within a common coordinate system.

Percutaneous needle biopsy illustrates these paradigms. Conventional CT-guided biopsies rely on repeated imaging to confirm needle placement, increasing procedure time and radiation exposure. MRI offers superior soft-tissue contrast and real-time visualization once the needle is within tissue, though initial targeting remains challenging. Ultrasound provides real-time guidance for superficial or well-visible lesions, highlighting the complementary nature of different imaging modalities [2, 3].

Navigation-based IGI systems enable patient-to-image registration and real-time instrument tracking using electromagnetic, optical, laser-based, or robotic technologies [4, 5]. However, electromagnetic systems are limited by magnetic interference, optical systems by size and MRI integration, and robotic systems by cost and limited MRI compatibility [6, 7].

Despite these challenges, optical tracking remains a versatile navigation technology due to its accuracy, modality independence, and cost-effectiveness. In this work, we present a Near-Infrared (NIR) monocular optical tracking system, based on a previously developed approach [8], for real-time navigation of percutaneous biopsy needles under CT and MRI guidance. The system uses retroreflective ArUco fiducial markers [9] to provide sub-millimetric accuracy, low latency, and reduced hardware complexity. Two experimental studies evaluated feasibility, accuracy, workflow efficiency, and MRI compatibility, including image quality and tracking performance in the MRI environment. The results support its potential as a low-cost, accurate, and MRI-compatible alternative for image-guided percutaneous interventions.

## Methods

**Fig. 1:**
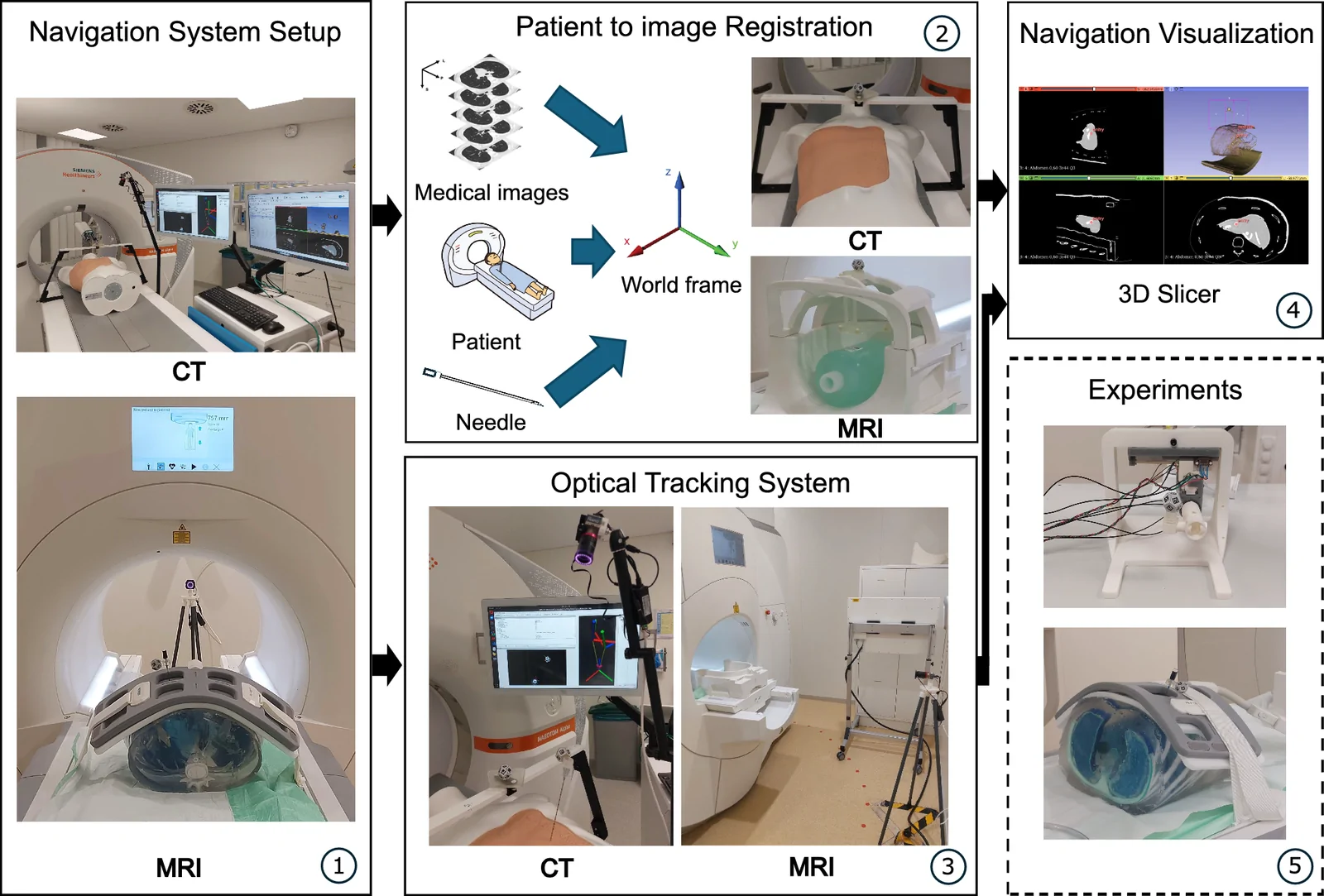
Block diagram of the CT- and MRI-guided needle biopsy navigation workflow. The system combines patient-to-image registration, monocular NIR optical tracking of the reference and needle markers, real-time transformation of the needle pose into the image coordinate system, and visualization of the planned trajectory and current needle position in 3D Slicer

Figure 1 shows the block diagram of the navigation system for CT- and MRI-guided percutaneous biopsy. Both setups share patient-to-image registration, optical tracking, and navigation visualization, enabling alignment of patient, imaging data, and instruments within a common coordinate frame.

Optical tracking is based on a monocular NIR camera and retroreflective ArUco fiducial markers [8], supporting registration and real-time needle tracking. Tracking data are streamed to 3D Slicer for planning and navigation. Subsequent sections describe the system components and experimental evaluation for each image modality.

### Navigation biopsy needle in CT

**Fig. 2:**
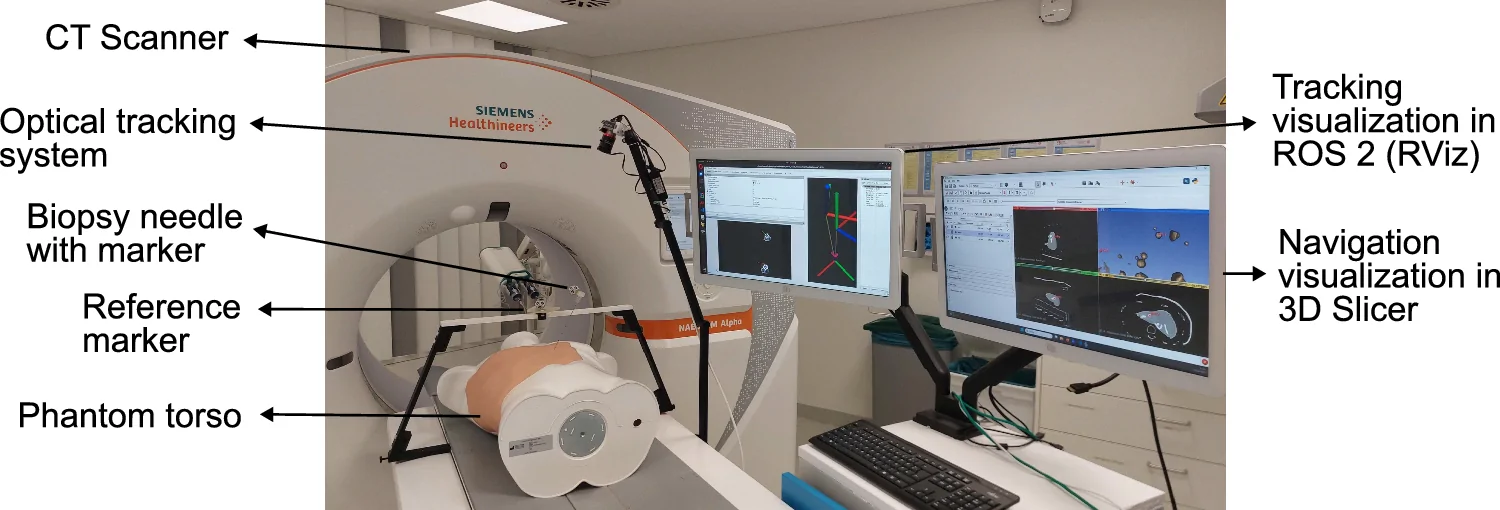
Setup for the CT-guided needle biopsy procedure

The CT-guided needle biopsy setup is shown in Fig. 2. It includes a table-mounted reference marker on a sliding 3D-printed support, a needle-mounted marker, and an optical tracking system rigidly fixed to the table side to remain stationary during table motion. Experiments were performed on a Quant® CT-Training Phantom (CAScination) using a Siemens Healthineers NAEOTOM CT scanner.

#### Patient-to-image registration

**Fig. 3:**
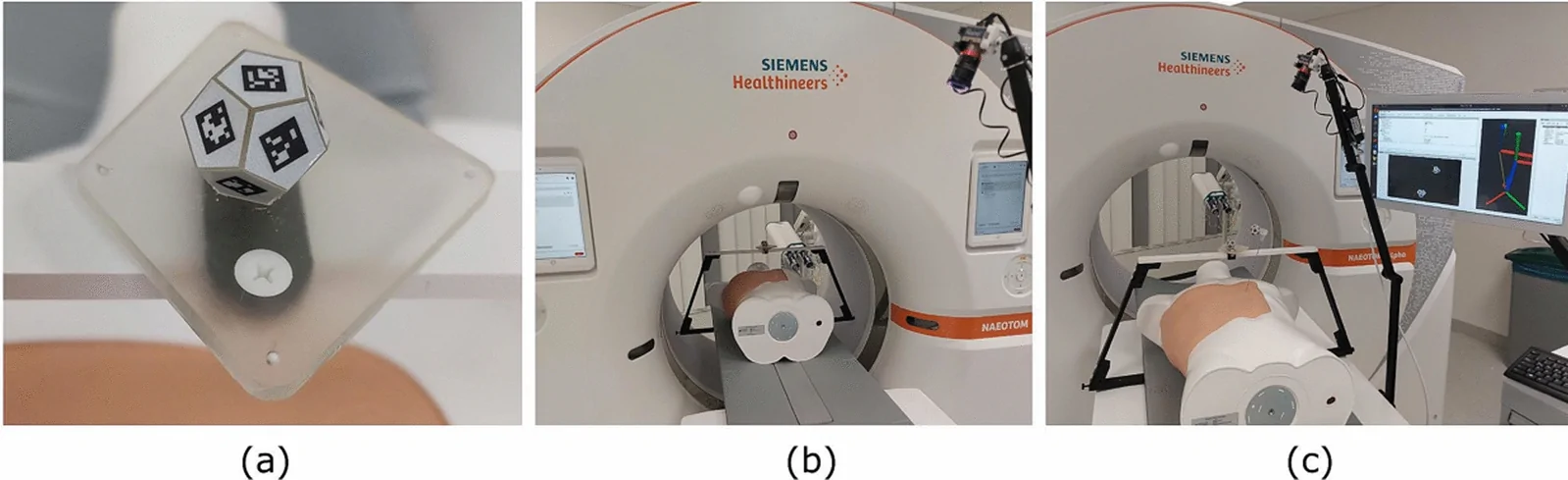
Registration setup: (a) dodecahedron-shaped marker with ArUco patterns and ZrO_2_ spheres; (b) support positioned inside the CT gantry during scanning; (c) support within the camera field of view for needle tracking relative to the CT coordinate system

The registration workflow begins by positioning the 3D-printed support on the CT table near the planned biopsy slice. A dodecahedron-shaped reference marker is mounted on the support, featuring ArUco markers on each face and four 2-mm-diameter ZrO_2_ spheres (precision grade G5) to ensure high CT contrast (Fig. 3a).

A CT scan is acquired to localize the spheres in the CT coordinate system. Sphere centroids are obtained in 3D Slicer via threshold-based segmentation and center-of-mass computation. Given the known sphere-to-marker geometry from the CAD model, the rigid transformation from the CT frame to the reference marker is estimated using the Umeyama algorithm [10], as expressed in Eq. 1. Three non-collinear fiducials are sufficient for point-based rigid registration. In the CT setup, four ZrO_2_ spheres were available, with one sphere providing redundancy for assessing registration accuracy.

1 $$\begin{aligned}  &   \textbf{T}_{\textrm{CT} \rightarrow \mathrm {ref\_marker}}\nonumber \\  &   \quad = \operatorname {Umeyama}\!\left( \{\textbf{p}^{\textrm{CT}}_i\}_{i=1}^{4},\; \{\textbf{p}^{\mathrm {ref\_marker}}_i\}_{i=1}^{4} \right) \end{aligned}$$

After the CT acquisition, the table is repositioned until the reference marker becomes visible to the optical camera. The needle-mounted marker is then introduced into the camera’s field of view. Its pose relative to the CT coordinate system is computed as:

2 $$\begin{aligned}&\textbf{T}_{\textrm{CT}\rightarrow \mathrm {needle\_marker}} = \textbf{T}_{\textrm{CT}\rightarrow \mathrm {ref\_marker}} \nonumber \\&\quad \cdot \left( \textbf{T}_{\textrm{cam}\rightarrow \mathrm {ref\_marker}}\right) ^{-1} \cdot \textbf{T}_{\textrm{cam}\rightarrow \mathrm {needle\_marker}} \end{aligned}$$

Here, $\textbf{T}_{\textrm{CT} \rightarrow \mathrm {ref\_marker}}$ denotes the transformation from the CT coordinate system to the reference-marker coordinate system obtained during registration, while $\textbf{T}_{\text {cam} \rightarrow \text {ref\_marker}}$ and $\textbf{T}_{\text {cam} \rightarrow \text {needle\_marker}}$ correspond to the reference-marker and needle-marker poses measured by the optical tracking system, respectively.

This formulation allows the needle marker, and consequently the needle tip after pivot calibration, to be localized with respect to both the CT images and the optical tracking system (Fig. 3a–c).

#### Optical tracking system

After registration to the imaging data and patient, the tracking system enables real-time navigation [8]. It comprises a monocular NIR camera with an infrared ring light for robust detection of dodecahedral markers bearing ArUco MIP_36_12 tags. The system achieves sub-millimetric positional and sub-angular rotational accuracy, is robust to ambient light and is compatible with CT and MRI environments. The hardware configurations for both modalities are summarized in the supplementary material.

**Fig. 4:**
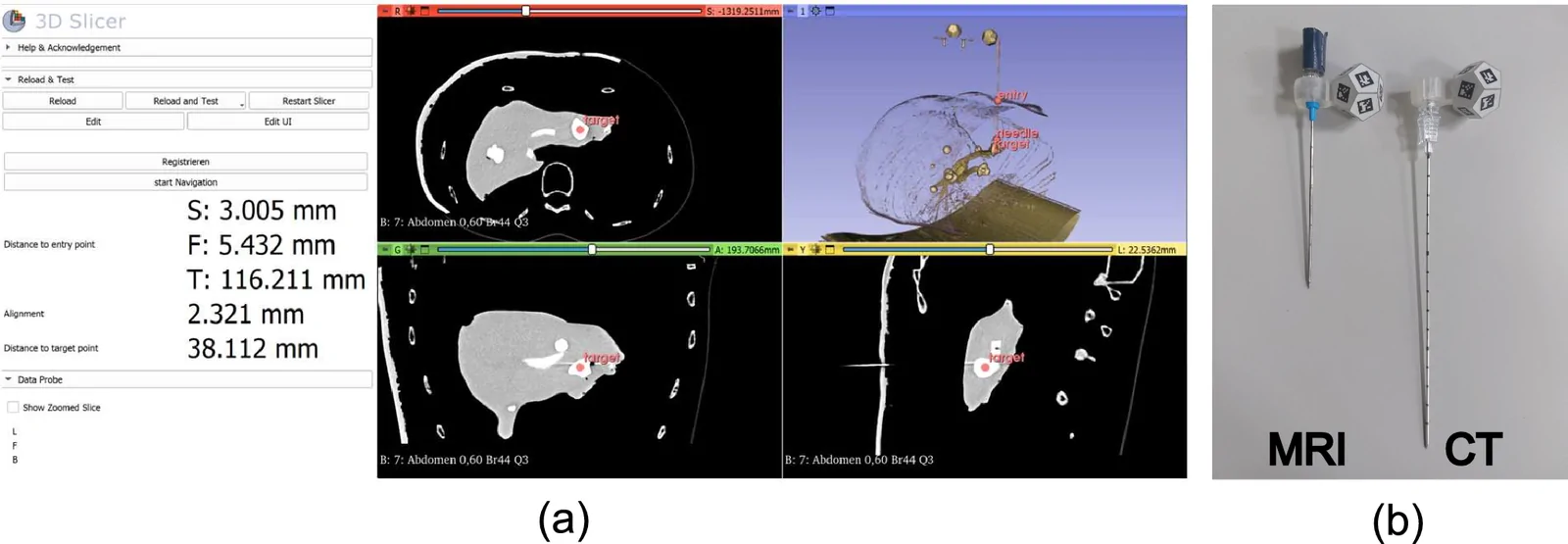
(a) 3D Slicer navigation module for real-time needle biopsy guidance. (b) Needles with markers used in the experiments during navigation for CT (right) and MRI (left)

The biopsy needle (C1820A Bard TruGuide, 17 G × 17.8cm - Fig. 4b) was equipped with a 3D-printed marker via a custom adapter (Fig. 4). A standard pivot calibration was performed to estimate the fixed offset between the marker and the needle tip, allowing accurate real-time tip tracking during navigation.

#### Navigation visualization

To facilitate intuitive use of the tracking data during CT-guided needle biopsy, a custom 3D Slicer module was developed using the IGTLink interface [11] (Fig. 4a). 3D Slicer provides a widely adopted open-source platform for image-guided interventions with real-time visualization and extensibility [12].

The module enables biopsy navigation by visualizing the needle in the CT volume and supports trajectory planning through user-defined entry and target points. The distances between the needle tip and the planned entry point along the sagittal, frontal, and transversal planes are displayed to assist initial positioning. Additionally, the deviation between the current needle trajectory and the planned path is shown to allow rapid alignment. During insertion, the remaining depth to the target is continuously provided.

### Navigation biopsy needle in MRI

**Fig. 5:**
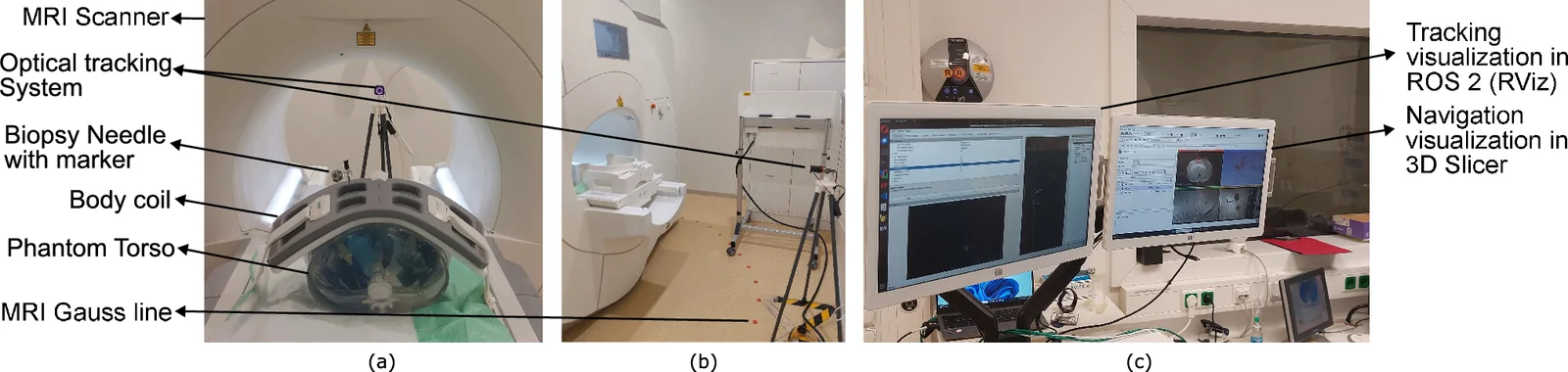
Setup for the MRI-guided needle biopsy procedure. (a) Front view of the MRI scanner. (b) Rear side of the scanner. (c) Control room

The setup for the MRI-guided navigation system (Fig. 5) is similar to the CT configuration, with some adaptations due to the restrictions on ferromagnetic materials within the gantry and its immediate vicinity. For this reason, the optical tracking system is positioned outside the MRI-compatible zone, at the rear of the gantry, and oriented to monitor the interior. Similarly, the biopsy planning and visualization system is located outside the MRI room.

For the experiments, an abdominal phantom with embedded MRI-compatible lesions [13] was used. Imaging was performed on a 1.5T scanner (MAGNETOM AERA, SIEMENS Healthineers).

#### Patient-to-image registration

**Fig. 6:**
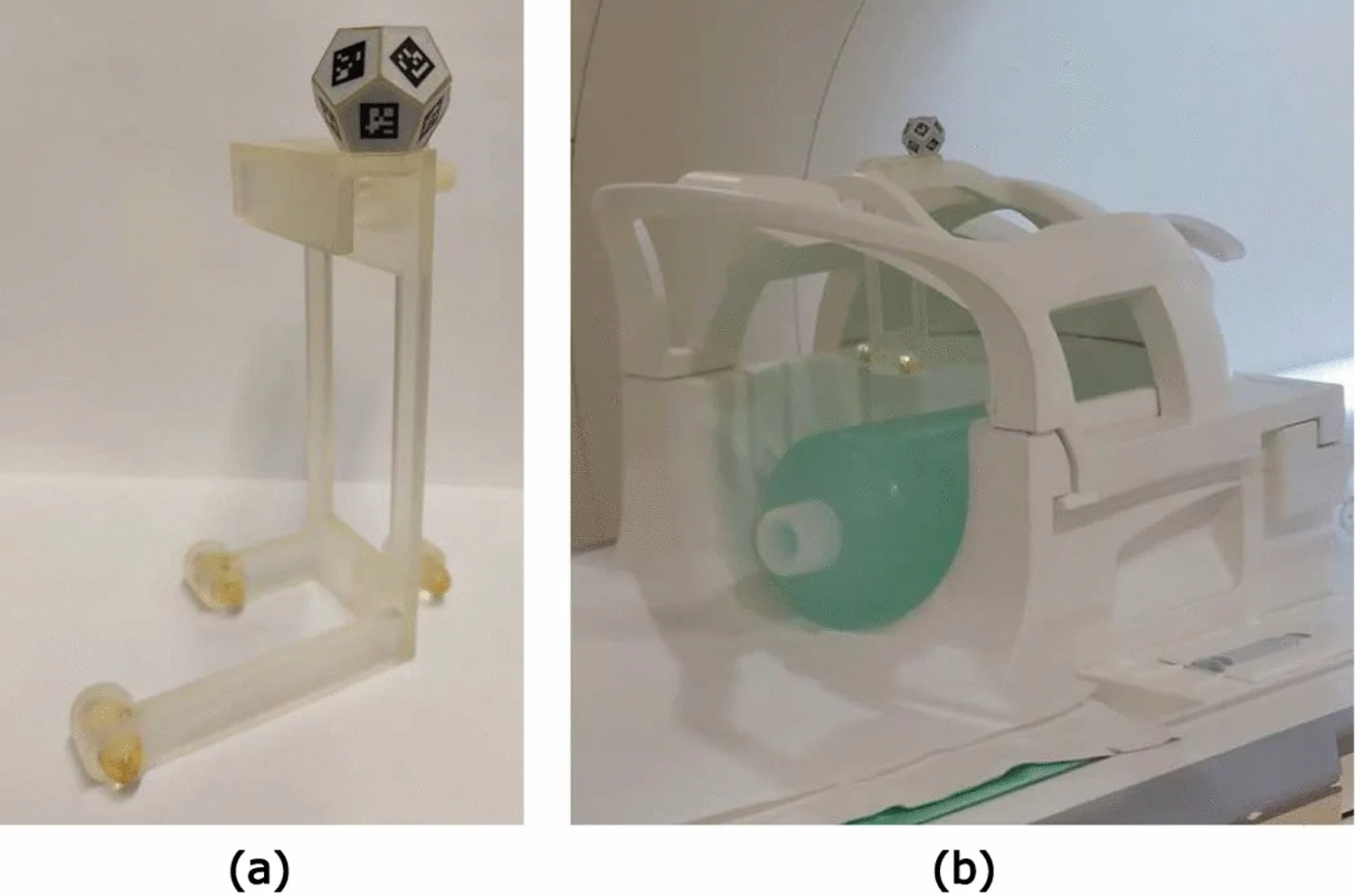
(a) Structure used for the patient-to-image registration, consisting of 3 omega-3 capsules and an optical reference marker. (b) Registration setup with the capsules placed inside the Head/Neck coil and the reference marker positioned outside the coil to ensure a clear line of sight to the camera

In contrast with the CT setup (Section Patient-to-image registration), the MRI registration system uses three high-contrast omega-3 capsules placed inside the 20-channel Head/Neck coil (SIEMENS Healthineers) together with a standard water phantom (SIEMENS Healthineers). A polyhedral reference marker is mounted externally to maintain line of sight with the optical camera (Fig. 6). After the reference MRI scan, the capsules were segmented offline in MATLAB using thresholding, and their centers of mass were computed in the MRI image coordinate system. The fixed capsule-to-marker geometry from the CAD model was then used to register the MRI coordinate system to the camera. The same CT-based registration equations (Eqs. 1 and 2) were then applied to MRI using three points, yielding the needle-marker pose relative to the MRI isocenter (Eq. 3). These three omega-3 capsules provided the minimum number of non-collinear fiducials required for rigid registration.

3 $$\begin{aligned}&\textbf{T}_{\textrm{MRI}\rightarrow \mathrm {needle\_marker}} = \textbf{T}_{\textrm{MRI}\rightarrow \mathrm {ref\_marker}} \nonumber \\&\quad \cdot \left( \textbf{T}_{\textrm{cam}\rightarrow \mathrm {ref\_marker}}\right) ^{-1} \cdot \textbf{T}_{\textrm{cam}\rightarrow \mathrm {needle\_marker}} \end{aligned}$$

It should be noted that, unlike CT-guided procedures, in this case the biopsy is performed with the patient inside the MRI gantry. During the intervention, the table—and therefore the patient—remains stationary relative to both the camera and the MRI isocenter. Consequently, the camera position with respect to the MRI isocenter remains constant, making the registration process a one-time operation rather than requiring repetition for each intervention.

#### Optical tracking system

The optical tracking system for MRI is analogous to that used in CT, but a different lens was selected to accommodate the altered workspace: the camera must be positioned outside the MRI gantry’s safety zone. The camera-to-isocenter distance is approximately 190 cm, with a practical workspace width of approximately 20 cm, covering the region around the isocenter.

A M1930 Teledyne DALSA Genie Nano-NIR camera (1920×1200 pixels) with a 50 mm C-series fixed focal length VIS-NIR lens was employed. The horizontal and vertical fields of view were calculated as 10.56$^\circ$ and 6.6$^\circ$, respectively, yielding approximately 36 pixels per side of an ArUco marker at 200 cm—sufficient for sub-millimetric accuracy [8]. To support dynamic marker tracking, the smallest aperture ensuring marker visibility (f/11) and short exposure times (<4 ms) were adopted [14], resulting in a depth-of-field range of 183–198 cm, sufficient to cover the MRI workspace given the algorithm’s tolerance to slight defocus.

The retroreflective ArUco marker was 3D-printed and attached near the tip of a 100 mm Interventional Cannula KIM-18/10T (ITP GmbH; Fig. 4b) for MRI-guided procedures. The position of the needle-tip relative to the marker frame was obtained using the same pivot calibration procedure described in Section Optical tracking system.

#### Navigation visualization

MRI navigation uses the same visualization as the CT setup, described in Section Navigation visualization.

### Experiments

**Navigation accuracy and workflow time in CT:** CT-guided needle biopsy was evaluated in a phantom model. A single CT scan was acquired and loaded into 3D Slicer for trajectory planning by selecting entry and target points (Section Navigation visualization). With continuous positional feedback from the tracking system, the needle was aligned and inserted to the planned depth, while procedure time was recorded.

To analyze the experimental results, two quantitative indicators, needle-tip tracking error (TTE) and target positioning error (TPE), were introduced. TTE was defined as the distance between the virtual needle-tip position provided by the tracking system and the needle-tip position in the post-insertion CT. TPE was defined as the distance between the post-insertion CT needle-tip position and the planned target position. The experiment was repeated ten times with different trajectories, recording procedure time, planned entry and target positions, and tracked and CT-derived needle-tip locations.

**Optical tracking evaluation in MRI:** The tracking system was assessed in a clinical MRI environment to evaluate possible interference from the static magnetic field and RF signals. A marker mounted on a motorized manipulator was moved to 100 positions in a 10×10 grid with 6.5 mm spacing. Encoder-based ground-truth positions were compared with tracking output outside the scanner room, inside the inactive scanner bore, and during real-time imaging (BEAT Multislice). All data were transformed into a common coordinate frame and positional deviations were computed.

**Navigation accuracy and workflow time in MRI:** MRI-guided accuracy and workflow time were evaluated using the CT protocol with modality-specific adaptations. An MRI-compatible phantom and shorter needle were used to reflect the shallower target depth. After insertion, two MRI scans were acquired, with the second plane aligned to the needle to facilitate tip segmentation. A turbo spin echo sequence (4-channel flex abdomen coil, Siemens Healthineers) was used (FOV 320×320 mm$^2$, matrix 336×336, flip angle $160^\circ$, slice thickness 3 mm). The first scan comprised 20 slices ($\textrm{TR}=4820$ ms) and the second 10 slices ($\textrm{TR}=2410$ ms). Each experiment was repeated ten times.

## Results

**Navigation accuracy and workflow time in CT:** The TPE and TTE were $10.3 \pm 3.7\,\textrm{mm}$ and $5.0 \pm 1.3\,\textrm{mm}$, respectively. The intervention time was $62 \pm 34\,\textrm{s}$.

**Optical tracking evaluation in MRI:** The RMSE between tracking measurements and manipulator encoders was $1.9\,\textrm{mm}$ outside the MRI room, $1.1\,\textrm{mm}$ in the static magnetic field, and $1.2\,\textrm{mm}$ during real-time imaging for static positions. For dynamic measurements, RMSE values were $3.3\,\textrm{mm}$, $1.9\,\textrm{mm}$, and $2.3\,\textrm{mm}$, respectively. The tracking update rate remained stable at approximately 70–72 Hz across all conditions. No degradation in tracking performance or MRI image quality was observed, indicating negligible electromagnetic interference.

**Navigation accuracy and workflow time in MRI:** The TPE and TTE were $4.6 \pm 1.0\,\textrm{mm}$, and $4.7 \pm 2.3\,\textrm{mm}$, respectively, with a mean procedure time of $113 \pm 65\,\textrm{s}$.

## Discussion

This study evaluated a previously developed optical tracking system [8] as a low-cost, multi-modality navigation tool for CT- and MRI-guided needle biopsy in phantom models, intended as an alternative to modality-specific and technically complex commercial platforms [15, 16].

A unified navigation pipeline with modality-specific adaptations was implemented. In CT-guided procedures, mean workflow time was approximately 1 min, with a mean final TPE of 10.3±3.7 mm. The dominant error sources included angular error amplification over needle length, pivot-calibration uncertainty, registration inaccuracies, marker segmentation, and table vibrations. Image-to-tracker registration contributed RMSE values of 0.6 mm for CT and 0.7 mm for MRI, calculated from image-segmented fiducials and their corresponding CAD-model positions. The slightly higher TTE observed in CT compared with MRI was mainly attributed to the longer CT needle, which amplifies small angular tracking and pivot-calibration errors at the needle tip. The larger CT TPE was further influenced by greater insertion depth, increased needle deflection, limited trajectory correction during insertion, and possible CT table motion after registration.

For the CT needle length of 178 mm and the previously reported angular tracking error of $0.35 \pm 0.16^\circ$ [8], the angular contribution to tip displacement is approximately $178 \cdot \sin (0.35^\circ ) = 1.09$ mm. Combined with the reported positional tracking error of 0.44±0.20 mm [8], this yields an ideal tracking-related tip uncertainty of about 1.18 mm. Thus, the larger navigation chain error of 5.0±1.3 mm is likely dominated by workflow-related factors such as pivot calibration, CT fiducial segmentation, image-to-tracker registration, and table-motion-induced displacement.

For MRI-guided procedures, the system preserved image quality and achieved low tracking errors under both static and dynamic conditions. Static RMSE values ranged from 1.1 to 1.9 mm, whereas dynamic RMSE values ranged from 1.9 to 3.3 mm, with only a moderate increase during motion. Procedure times were approximately 2 min. The improved MRI-guided TPE of 4.6±1.0 mm compared with CT was attributed to the shorter needle and stationary reference configuration [17, 18].

The MRI-guided TPE of 4.6±1.0 mm is comparable to values reported for state-of-the-art image-guided liver interventions, including MRI-guided liver biopsy with median lateral deviations of approximately 3.4 mm and 96% diagnostic yield [19], stereotactic CT-guided liver biopsy with median needle-tip errors of approximately 3.2 mm and 97.8% diagnostic yield [20], and experimental optical CT navigation with mean errors around 3.5 mm [21].

In contrast, the CT-guided experiments resulted in a larger TPE of 10.3±3.7 mm. Although sufficient for the present phantom experiments, this accuracy is lower than that of advanced stereotactic or robotic systems and may be insufficient for reliable clinical targeting of small liver lesions of approximately 10 mm diameter without further workflow and mechanical optimization. Additional mechanical stabilization and workflow optimization are therefore needed before clinical translation.

A further limitation is that all experiments were performed in static phantoms without respiratory motion. In clinical liver interventions, respiration, patient motion, tissue deformation, and variable lesion visibility may introduce additional errors. Nevertheless, the MRI-guided accuracy remained within the range reported in previous clinical liver intervention studies [19, 20]. Future studies should evaluate dynamic conditions using motion phantoms, breath-hold protocols, respiratory gating, or motion compensation strategies.

Another limitation is the need for a clear line of sight between the camera and tracked markers, especially in MRI because of the limited space near the scanner bore. In our setup, the camera was placed at the rear side of the scanner, and the intervention was mainly performed from the opposite side to reduce marker occlusion. However, clinician movement or instrument handling could still interrupt tracking. Future implementations could use an MRI-compatible camera positioned inside or closer to the bore to improve robustness.

Overall, the results support the feasibility of optical tracking as a low-cost solution for multi-modality percutaneous interventions, while highlighting the need for further optimization in CT-guided workflows.

## Conclusion

This work demonstrated a low-cost optical tracking-based navigation system for CT- and MRI-guided needle biopsy. The system achieved MRI-guided navigation accuracy comparable to previously reported optical and stereotactic navigation approaches, while the CT-guided setup demonstrated feasibility but requires further optimization to improve targeting accuracy for small lesions.

The proposed approach provides a flexible, modality-independent framework with reduced technical complexity and cost, improving accessibility for image-guided interventions. Its MRI implementation is particularly noteworthy given the limited availability of compatible navigation systems.

Limitations include the phantom-based evaluation, manual needle manipulation, limited sample size, registration uncertainties, absence of respiratory motion and tissue deformation, and the line-of-sight requirement of the optical tracking system. Future work will focus on mechanical stabilization, improved registration robustness, validation under dynamic and more clinically realistic conditions, usability testing with medical personnel, comparison with standard free-hand workflows, and extension to ultrasound-guided procedures.

## Supplementary Information

Below is the link to the electronic supplementary material.

Supplementary file 1 (docx 27 KB)

## Data Availability

Available from the corresponding author upon reasonable request.

## Code availability

Available from the corresponding author upon reasonable request.
