# Supplementary material for: Implementation of monocular near-infrared camera tracking in MRI and CT navigation systems
Source: Int J Comput Assist Radiol Surg. 2026 Jul 30;21(11):2231–8. doi: 10.1007/s11548-026-03758-1 (PMC13759625; doi:10.1007/s11548-026-03758-1)
Supplement: Supplementary file 1 [file 11548_2026_3758_MOESM1_ESM.docx]

**Supplementary Material**

**Optical Tracking Hardware Specifications**

*Camera, lens, and light specifications of optical tracking system for CT and MRI.*

| **Component** | **Feature** | **Value** |
| --- | --- | --- |
| **Camera** | Type | NIR, Monochromatic |
|  | Sensor size | 9.22 x 5.76 mm |
|  | Exposure Time | 0.5 ms (CT), 4.0 ms (MRI) |
|  | Resolution | 1920 x 1200 |
|  | FPS | 80 |
|  | Analog Gain | 0 dB |
| **Lens** | Focal Length | 16 mm (CT), 50 mm (MRI) |
|  | f-stop | 5.6 (CT), 11 (MRI) |
| **Light** | Wavelength | 875 nm |
|  | Power | 2 x 3 W |
|  | Type | Directional Ring |
